# Drones in ecology: ten years back and forth

**DOI:** 10.1093/biosci/biaf069

**Published:** 2025-06-19

**Authors:** Karen Anderson, Felipe Gonzalez, Kevin J Gaston

**Affiliations:** Environment and Sustainability Institute, University of Exeter, Penryn, Cornwall, England, United Kingdom; Robotics and Autonomous Systems, Queensland University of Technology, Brisbane, Queensland, Australia; Environment and Sustainability Institute, University of Exeter, Penryn, Cornwall, England, United Kingdom

**Keywords:** drones, spatial ecology, survey, sampling, aerial

## Abstract

A decade after our initial publication predicting that lightweight drones would revolutionize spatial ecology, drone technology has become firmly established in ecological studies. In the present article, we explore the key developments in ecological drone science since 2013, considering plant and animal ecology, imaging and nonimaging workflows, advances in data processing and operational ethics. Focusing on inexpensive, lightweight drones equipped with various sensors, we offer a critical evaluation of drone futures for ecologists, arguing that this could deliver opportunities for volumetric ecology to take flight. We discuss the potential future uses of drones in aerobiology and in understory and underground ecological studies and debate the future of multirobot cooperation from an ecological standpoint. We call on ecologists to engage critically with drone technology in this next phase of development.

Ten years ago, we asserted that lightweight consumer-grade drones (i.e., unpiloted aerial vehicles) would revolutionize spatial ecology (Anderson and Gaston 2013). Prior to 2013, the aerial vantage point was hard to access; subsequently, affordable drones (as flying cameras, initially) created a means of new aerial experimentation by ecologists. In that original article, we summarized the different types of drones and classified them by size or takeoff mass (TOM). We underlined a series of technical and applications-based considerations, highlighting areas that could be of interest to ecologists. Some of the research fields that we proposed as ripe for ecological exploration have mushroomed in the past decade, others less so. We refer readers to table 1 for a summary retrospective on the key areas highlighted in 2013 and their developments over the past decade. It is certainly true to state that huge developments have been achieved in a decade; fueled by a need to explore the role of the environment in modulating organismal traits, abundances and distributions, ecologists have adapted this aerial toolkit in creative ways. Some advancements were easily foreseen (e.g., drones in conservation and for field studies of wildlife; Wich and Koh 2018 and Corcoran et al. 2021b, respectively). Others were less easily predicted and have emerged as drone and sensor technologies have evolved rapidly and in tandem over the past decade. The present article has two sections. The first section provides a critical retrospective on what is now more than a decade's worth of ecological experimentation with drones. Second, we highlight and discuss potential new avenues of research for drone ecology, suggesting how the next decade of experimentation by ecologists will shift further into the volumetric (i.e., three-dimensional [3D] sampling or sensing) domain.

## Drone ecology 2013–present

A comprehensive review of over a decade's research^1^ is beyond the scope of this article. Instead, we direct readers to other reviews of drone technology within subfields of ecology (i.e., wildlife research and management, Chabot and Bird 2015; organismal counting from drone image data, Chabot and Francis 2016; forest health monitoring, Ecke et al. 2022; forestry management practices, Tang and Shao 2015; marine monitoring, Yang et al. 2022). Summarizing and classifying the major advancements, table 2 highlights the range of drone-based work now routinely carried out in spatial ecology. Although our review is not exhaustive, the cited articles linked in the table will give readers information about toolkits and technical workflows via worked examples. The diversity of journals within which these papers appear is a testament to the interdisciplinary nature of drone ecology.

Drone ecology looks very different now from how it did a decade ago. The convenience and the relatively low financial cost (a few hundred to a few thousand US dollars) have made commercial drones available to ecologists. Compared with self-build drone systems, which were commonplace 10 years ago, the current generation of ready-to-fly systems are vastly more reliable, featuring power fail-safes, automatic takeoff and landing, and major obstacle avoidance. Furthermore, the growing commercial dominance and straightforward operational capabilities of aircraft from the drone manufacturer DJI (who controls 70% of the commercial drone market), together with their influence over global drone legislation, have potentially contributed to self-build models falling out of favor in the scientific realm. This is despite the sometimes reduced financial cost of self-build components and batteries and the open nature of DIY platforms such as Pixhawk and Mission Planner being more aligned with open science.

**Figure 1.**
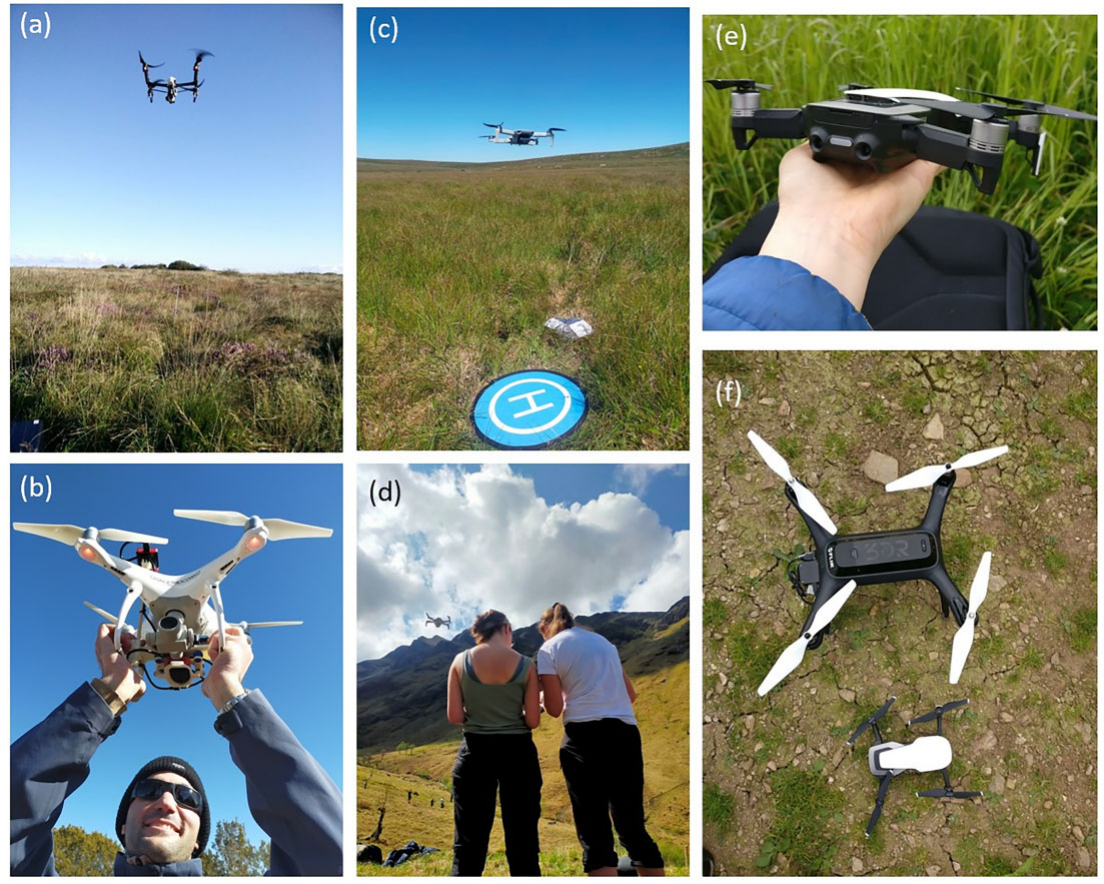
Examples of lightweight drones being used for ecological survey. (a) DJI Inspire 1 (take-off mass [TOM]: 2.93 kilograms [kg]); (b) DJI Phantom-4 pro (TOM: 1.38 kg) equipped with an additional multispectral sensor (Parrot Sequoia); (c) DJI Mini-2 (TOM: 242 grams [g]) with a ground-control marker and landing pad shown; (d) DJI Mini-2 (TOM: 242 g) being flown by undergraduate students during a field class; (e) DJI Mavic Mini (TOM: 249 g) shown in an adult hand for scale, and (f) beside a 3D Robotics Iris+ (TOM: 1.28 kg) for comparison. All images author's own (KA).

Consumer-grade drones have become popular ecological tools because they are easy to fly and because aviation policy permits them to be used in all but the most congested spaces (the rules vary internationally; Cracknell 2017, Cunliffe et al. 2017, Tsiamis et al. 2019, Hodgson and Sella-Villa 2021). Camera-equipped drones with the lowest takeoff weight (e.g., DJI mini; TOM < 250 grams; figure 1c–1f) are classified as toys in most countries and require no pilot certification to fly them, but some have good cameras and the capability to follow GPS-guided flight plans. These tiny backpack-carried drones can be operated in areas that are off limits for larger or heavier drones.

**Table 1.** Key points raised in Anderson and Gaston (2013) and notes on how ecological developments have responded to those suggestions.

In 2013 we wrote…	What happened…
Highly miniaturized micro-UAVs could “provide valuable data for ecological studies, such as insights into animal behavior in environments where human observers and larger UAVs would otherwise disturb the subject organism.”	Within the ecological realm this has not yet been proven widely but new innovations in this class of very small drones have come to the fore since 2013. It seems that the influence is more from ecology to drone engineering rather than the other way around, for example, in drone or sensor design being biologically inspired. Ecologists and drone technologists should work closely together from this point forwards to explore synergies in the applications realm.
Work is needed to ensure radiometric quality of drone-captured data.	This area has developed considerably with new studies emerging in recent years that speak to the robustness of the remote sensing signal. In particular we highlight work by the group based in the Finnish Geospatial Research Institute who have undertaken a wealth of basic experiments testing the robustness of physical measurements such as canopy reflectance from drones.
Care must be taken to mitigate vibration effects and impacts on data quality.	For most systems now this can be successfully ameliorated or mitigated via stabilized sensor gimbals. Most commercial systems are capable of sophisticated three-axis sensor stabilization in this way. The days of using homemade payload holders with piano wires or blue gel to reduce vibration transfer to sensors is over.
Geometric stability and accuracy as a data quality concern.	Huge leaps forwards have been made in workflows that use ground control markers, such that this is a proven methodology widely used for calibration and geometric validation. More recently there has been the emergence of fully integrated Global Navigation Satellite System (GNSS) on the drone with real-time kinematic (RTK) relay to a ground base station for improvement of geometric accuracy. Systems equipped with RTK-GNSS are more costly to buy than those without.
“Lightweight UAVs are challenging to pilot.”	This concern has virtually evaporated for most lightweight drones now with huge improvements in pilot control systems and fail-safes. Operating heavier drones remains a highly skilled, and potentially stressful job, because of operational risks with heavier aircraft, more involved paperwork for operational approval from aviation authorities, and the increased financial risk of loss if something goes wrong.
“Site conditions, such as dense tree canopy cover, may make real-time tracking difficult or prevent platform retrieval if the UAV goes off track.”	The wealth of studies where drones have been used in diverse settings has enabled a great breadth of experiences and methodological recommendations to be made. Understanding of the constraints of the technology in specific location-based settings is now well scoped.
Thermal imaging will offer benefits for monitoring nocturnal species.	This is now a relatively well-tested approach within ecology, with the caveat that thermal sensors suffer from reduced spatial resolution compared with optical counterparts. Some off-the-shelf commercial thermal drone systems are now available for purchase as out-of-the-box options.
Objects or organisms in drone images were mostly manually counted in 2013. We said that including pattern recognition would boost information extraction.	Machine learning tools are now mature and widely used for such applications, including tracking of individuals in drone captured video recordings. Table 2 gives some examples.
In vegetation studies we identified that new sensors with infrared capabilities could revolutionize retrieval of key vegetation parameters.	This approach is now mature, although, in 2013, it was an emerging possibility. Off-the-shelf drone systems with multispectral RGB and IR capabilities are widely available now. There are other bespoke multispectral or hyperspectral sensors available for attachment to drones. Ecologists use these techniques widely. Standard and widely used vegetation indices such as NDVI are often generated from the raw data. Regular calibration of sensors is a requirement.
We wrote one sentence about LiDAR for drones, which wasn't widely explored in 2013, because of weight constraints and the size of LiDAR instruments at the time.	Drone LiDAR is now operational and beginning to be used within ecological studies, particularly by vegetation and landscape ecologists. The sensor technology and drones to carry them have only emerged in affordable formats in the past 5 years or so. Major drone manufacturers now offer LiDAR equipped systems.
Structure from motion photogrammetry was emerging as a potential area of interest in 2013. We said it could provide efficient and low-cost workflows for vegetation structural assessment.	This is now commonplace as an approach within spatial ecology, because low-cost camera-equipped drones are commonplace (being cheaper to buy and easier to operate compared with LiDAR drones). There are limits to use particularly in coalesced canopies, and accuracy of canopy height retrieval can also be affected by conditions such as windthrow in taller canopies. Ground control for geometric calibration and validation has proven to be essential.
Meteorological measurements from drones and the relevance to ecology was highlighted.	This remains an underexplored area compared with other fields that were highlighted in our 2013 paper. Drones have certainly been exploited within meteorological studies, but these techniques have been embraced less frequently by ecologists. Ecologists and biologists have instead chosen other attachments to drones that we did not foresee in 2013, for example, petri dishes for biological sampling and devices such as stem cutters for ecological sampling in hard to reach places. See figure 3 and associated text.

**Table 2.** Major areas of advancement in drone spatial ecology over the past decade.

Use case	Crucial aspects of drone workflow	Published examples
Mapping and baseline site surveys	Digital photogrammetry tools for converting 2D image sets into base maps (orthomosaics) and volumetric point clouds (e.g., canopy structural models) are now mature. Commercial or free (e.g., www.geonadir.com) workflows for data processing are available. Enhanced information may be provided by multispectral or hyperspectral imagers. Drone LiDAR delivers additional information about canopy structural attributes.	de Almeida et al. 2020, Robinson et al. 2022
Image-based counting of plant and animal populations	Drones are flown at particular heights to achieve optimal spatial resolution for the target species. For plants, improved discrimination may be achieved with near infrared sensors or by including structural information from photogrammetry or LiDAR. Drone-derived counts are reportedly more accurate than *in situ* counts (Hodgson et al. 2018). Strong drive toward machine-learning approaches for information retrieval.	Chowdhury et al. 2022, Oldeland et al. 2021, Popović 2020
Sonic counting of animal populations (e.g., bats and songbirds)	Bioacoustics sensors are carried aerially to capture data among the target organisms. Drone rotor noise must be filtered. Disturbance effects and ethics require careful consideration.	Wilson et al. 2022
Cryptic or arboreal species counts (e.g., mammals and birds)	Thermal infrared imagers commonly used, and organism detection is usually improved nocturnally in such cases. Species ID from thermal data alone remains a challenge. Machine learning workflows can improve detection rates when animals are partially obscured.	Corcoran et al. 2021b, McCarthy et al. 2021
Fruit and flower counting in varied settings	Widely tested in agricultural settings, some uses in ecology. Typically requires spectral and textural information and machine learning workflows. Computationally expensive, more efficient if executed on the cloud.	Gallmann et al.^ 2021^
Burrowing or nesting species distribution and counts (e.g., crabs, turtles, wombats)	Requires high-quality orthomosaics or photogrammetric/LiDAR-derived surface models to highlight microtopographic disturbances associated with burrows or nests.	Old et al. 2019
Biodiversity surveys	Spatial resolution is a key parameter because accurate information on shape, size, and sometimes spectral properties of objects may be crucial for accurate mapping. A range of sensors from passive optical to active LiDAR approaches may be used.	Getzin et al. 2012
Vegetation status, precision agriculture and phenology	Requires either a fully radiometric survey or relative indices derived from specialized sensors (e.g., infrared or narrow-band capabilities). Radiometric survey has an added requirement of a calibration stage against standard panels. Fully hyperspectral examples are less frequently used because of higher sensor costs, heavier payloads and complexities of data processing.	Dorin et al. 2023, Fawcett et al. 2020
Allometric or morphometric measurements of plants and animals	Can be performed with photogrammetry workflows or using drone-mounted LiDAR. Numerous studies in plant allometrics, with some breakthroughs in marine vertebrate morphometrics with drone photogrammetry. Drone-mounted LiDAR data have proven useful for plant branch and stem structure measurements. Airborne laser scanning approaches for canopy attribute retrieval can be applied to drone-captured, photogrammetry-derived point clouds.	Bierlich et al. 2021, Hodgson et al. 2020, Karl et al. 2020, Toivonen et al. 2021
Animal behavior, particularly social interactions and individual or group behavior	Typically uses videographic data. May use advanced geocomputational workflows, machine learning, or manual labelling and interpretation of footage. Platform stabilization is important. In marine mammals, key parameters such as swimming alignment, nearest-neighbor distances, velocity and tail beat frequency can be measured. Disturbance effect of the drone on target organisms needs careful consideration.	Butcher et al. 2021, Torney et al. 2018
Antipoaching operations	Various workflows used. Some exploit the drone's disturbance effect to move species away from risky areas to reduce poaching risk. Also see quasimilitary approaches where drones survey human subjects rather than the nonhuman species that are under protection, termed *green militarization* (Lunstrum 2014).	Penny et al. 2019
Human–wildlife conflict	Still relatively in infancy. For example, drone image data have been used to track movement and swim paths in white sharks close to popular beaches in Australia with a goal of shark bite mitigation and improving conservation outcomes. Motivated by wider work showing how understanding dynamic movement in ecology can underpin wildlife conservation (Fraser et al. 2018).	Colefax et al. 2020
Mammal and plant physiology	Airborne infrared thermography from drones can permit exploration of patterns of heat loss, providing insight into physiological mechanisms producing these patterns. So far has been trialed in the study of large mammals (whales, primates) and for parameterizing models to predict evapotranspiration from plants.	Ellsäßer et al. 2021, Horton et al. 2019, Lonati et al. 2022, Zhang et al. 2023
Restoration ecology	Highly experimental. Drones may be used to drop seeds on a restoration site (e.g., for afforestation). Evidence suggests that much of the drone-dropped seed may fail to germinate. Most operators are commercial rather than scientific. Humanities scholars have dubbed this a force of “technoliberalism” (Fish 2022b).	Castro et al. 2023
Telemetry applications	Telemetry sensors to pick up very high frequency (VHF) radio signals from tracked animals can be mounted on drones. There are numerous studies demonstrating the proof of concept (biases toward large species in more open habitats). Drone telemetry can deliver high detection rates from low search efforts for settled individuals, even in challenging ecological settings with small species (Stone et al.^ 2024^, Yamato et al.^ 2023^). Optimizing antenna orientation is crucial. Some open source systems have been described (Shafer et al.^ 2019^). Cost and time savings on ground tracker time have been reported with drone telemetry.	Tremblay et al. 2017 and see the special issue in *Wildlife Research* prefaced by Aaron et al. 2022

### Flying cameras for ecological survey

Lightweight consumer-grade drones (figure 1), carrying standard optical cameras and weighing less than 2 kilograms typically, have been the mainstay of many ecological experiments over the past decade. Most of these camera drones are not built with the scientific market in mind but can be readily adapted for scientific work (Chabot and Bird 2015). This technology has delivered a wealth of new scientific discoveries (tables 1 and 2); for example, drone photography enabled discovery of a new species of shrub on a remote canyon wall in Hawai'i (a drone was also used to capture a herbarium and seed sample; Wagner et al. 2024), revealed the lesser-known feeding behavior of sharks on whale carcasses (Tucker et al. 2019), and enabled submerged reef systems to be passively imaged, delivering unique insights into coral habitat structures even through disturbed water surfaces (Chirayath and Earle 2016, Chirayath and Instrella 2019). Other sensor types have emerged over recent years with multispectral cameras becoming integrated into drone ecology workflows, expanding sensing capabilities into near infrared regions of the spectrum—beneficial for vegetation surveys (note the work of Assmann et al.^ 2018^, who were careful to highlight the importance of sensor calibration and sensitivity of derived metrics to illumination conditions). Similarly, some off-the-shelf drone systems are now available with integrated thermal imagers, for measuring emitted thermal radiation. Tested by ecologists in various settings, drone thermography has proven particularly useful for nocturnal wildlife applications (Larsen et al. 2023) in arboreal ecology for, for example, surveys of koalas (Witt et al. 2020) and abundance estimation and behavioral studies of gibbons (Zhang et al. 2020). Combined thermal and visible camera surveys have proven useful in areas where habitats are challenging to reach and where visibly cryptic species are thermally distinct from their surroundings (e.g., in marsh bird surveys; McKellar et al. 2021).

### Volumetric data products from overlapping 2D image sets

Developments in software and data processing approaches during the past decade have fueled a volumetric turn within drone ecology. Foremost, computer-vision approaches used widely for digital photogrammetry have revolutionized the pipeline for production of orthomosaics, base maps, and volumetric point clouds from basic 2D aerial photographs captured by drones (D'Urban Jackson et al. 2020). This field has evolved rapidly since early work demonstrating its power for vegetation canopy structural modeling (Dandois and Ellis 2010, 2013) and has now been extended to address a broad range of ecological questions particularly in plant science (e.g., plant height measurement, Niu et al. 2024; aboveground biomass estimation, Cunliffe et al. 2016, Cunliffe et al. 2022, Harrison et al. 2024; forage use, Gillan et al. 2019) but has also informed ecological assessment (Spreitzer et al. 2022), restoration and management (Mestre-Runge et al. 2023, Nuijten et al. 2024), and the study of organismal morphometrics (Hirtle et al. 2022, Irschick et al. 2022). Volumetric information on the morphometry of fluvial and shallow submerged aquatic environments is also potentially achievable from drone photogrammetry workflows (for a review, see Carrivick and Smith 2019). Such techniques have been successfully demonstrated for, for example, coral reef rugosity monitoring (Casella et al. 2017, Casella et al. 2022)—a crucial indicator of reef complexity and habitat quality. Technical considerations differ from those in terrestrial applications; for example, there may be geolocation issues arising from lack of fixed ground control (Watts et al. 2024) and uncertainties arising from water surface disturbances (e.g., refraction; Casella et al. 2022) or periodic changes (tides or water depth), which can lead to modeling distortions if not corrected. In all such data pipelines, ecologists have had to refine data acquisition and processing workflows (i.e., including but not limited to the physical flying of the drone). A firm understanding has now emerged that *how* data are captured and processed influences the accuracy and reproducibility of resulting products (Forsmoo et al. 2019, Scher et al. 2019, Whitworth et al. 2022, Slade et al. 2024). Ecologists have guided much of the methodological innovation for generating high quality point clouds from 2D image sets (e.g., through protocols such as the High Latitude Drone Ecology Network (https://arcticdrones.org/protocols/) and other protocols (e.g. Cunliffe and Anderson 2019), but technical know-how on optimizing data capture and refining photogrammetric pipelines has also emerged from other disciplines particularly the geosciences (James et al. 2019). Fundamentally, ecological systems are inherently harder to model photogrammetrically than are bare Earth systems, because of tendencies toward subject mobility (e.g., plant movement by wind, Frey et al. 2018, Cunliffe et al. 2022, Slade et al. 2024) and because of the impact of changing illumination conditions and seasonal phenology on accuracy (Brown et al. 2024), and in coalesced plant canopies, there are limits to allometric retrieval because of feature occlusion (Wallace et al. 2016).

### New specialized drone sensors

**Figure 2.**
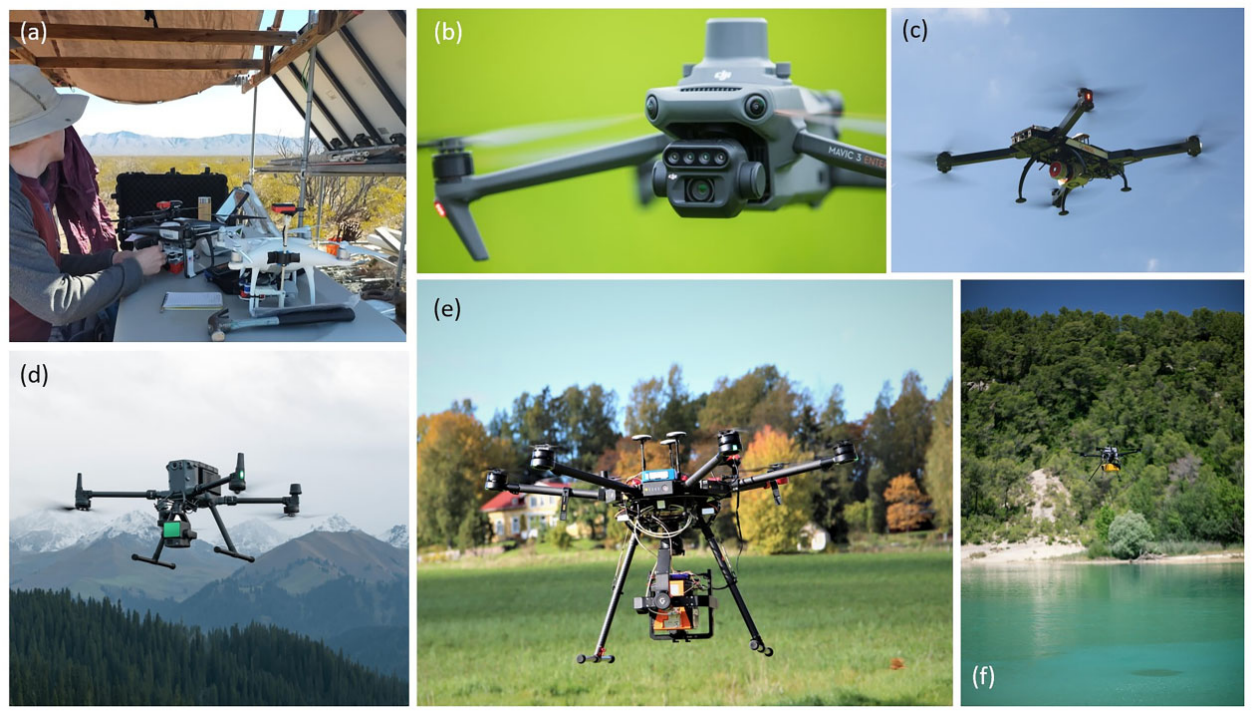
New sensor hardware that has emerged in the past decade includes (a) readily deployable multispectral instruments such as the Parrot Sequioa (foreground) and MicaSense Red Edge (background); these can be easily configured to be flown on standard consumer-grade drones (shown are DJI-Phantom type aircraft); (b) DJI's off-the-shelf multispectral drone (the “Mavic 3M”) with green, red, red edge, and near infrared sensors for measurement of vegetation spectral features (image: DJI); (c) RIEGL's RiCOPTER—a ready-to-fly drone LiDAR equipped with VUX-1UAV Lidar Sensor and integrated cameras (image: ©RIEGL, www.riegl.com); (d) DJI's Zenmuse L2 LiDAR system mounted on a Matrice drone platform (image: DJI); (e) Specim AFX10 VNIR sensor (400–1000 nanometers [nm], spectral resolution 5.5 nm, 224 bands, 1024 spatial pixels) onboard the DJI Matrice 600 Pro hexacopter drone (from Huo et al. 2024; image: National Land Survey of Finland); (f) Yellowscan Navigator bathymetric LiDAR sensor (a green laser with waveform capabilities), being flown on a Tundra-2 multirotor platform (image: Yellowscan).

Drone sensor hardware has advanced rapidly over the past decade (figure 2). Vegetation scientists in particular have capitalized on new compact multispectral and hyperspectral imagers for monitoring plant traits at a fine scale (Assmann et al. 2018, Hartfield et al. 2022, Huo et al. 2024). Drone-based light detection and ranging (LiDAR) has also developed at pace with numerous systems (both terrestrial and bathymetric) now available at much cheaper price points than they were a decade ago (tens of thousands of dollars, compared with hundreds of thousands previously; Kellner et al. 2019). The high price point still places drone LiDAR beyond the reach of many, plus the operational situation is more complex and heavily legislated owing to the increased takeoff mass of LiDAR-equipped drones (more than 10 kilograms typically) compared with standard consumer-grade photography drones. Despite this, the volumetric point clouds generated from drone LiDAR have value in vegetation studies (Getzin et al. 2022) and in forest mensuration work (Brede et al. 2017, Wieser et al. 2017) and have been demonstrated to deliver superior ecological information than airborne (i.e., piloted aircraft–mounted) LiDAR where small plants are the focus (Resop et al. 2019).

### More than aerial photography

**Figure 3.**
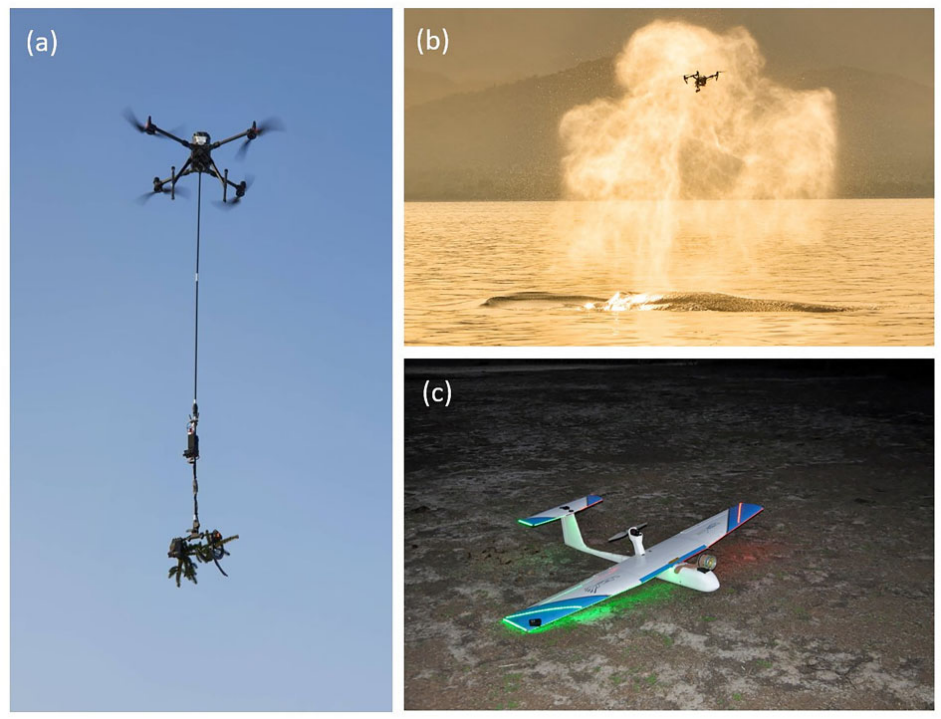
Novel drone platforms with nonimaging ecological capabilities. (a) The commercial company Biodrone uses aerial multirotor drones to take samples from upper branches of trees for DNA analysis (image: Atilla Haugen, biodrone.no). (b) Drone sampling whale sputum from blowhole mist using the snotbot—a drone fitted with Petri dish payload (image: Christian Miller and whale.org; Geoghegan et al. 2018). (c) An aerial insect sampler fitted to a fixed-wing drone (image: Margarita Mulero Pazmany, Doñana Biological Station CSIC, Spain; Mulero-Pazmany et al. 2022).

Drones offer ecologists an agile means of sampling in otherwise inaccessible places. Crucially, they permit monitoring aerially, among rather than vicarious to target organisms. Drones have been trialed as platforms for remote sampling of DNA—for example, taking cuttings (figure 3a; see also Wagner et al. 2024) or perching to capture eDNA samples using adhesive surfaces (Aucone et al. 2023). Entomologists can now fly among insect swarms (figure 3c; Mulero-Pazmany et al. 2022), while snotbot drones capture sputum samples from whale blowholes for biological analysis (figure 3b; Geoghegan et al. 2018). This allows for intimate human–wildlife interactions that are less violent and more ethically justifiable than methods previously used (e.g., blubber sampling for microbiome analysis; Fish 2022a). Such experiments would otherwise be technically difficult and time consuming to achieve. Keeping sight of the payload limits is important—in particular, aquatic sampling approaches may necessitate use of drones with larger payload capacities (Lally et al. 2019).

### Drone workflows versus traditional field sampling

An obvious use of drone-captured imagery is for population studies and beneficially, drone-derived population counts have been shown to deliver more accurate data than standard methods (Hodgson et al. 2018), with reduced survey costs (Old et al. 2019), although there are considerable costs associated with computational information extraction *post hoc* (e.g., Tay et al. 2018 commented that challenges of image processing lengthen the drone workflow compared with traditional sampling). Drone data analysis is a “big data” discipline, and there has been an emergence of machine- and deep-learning approaches applied to drone data (e.g., Gallmann et al. 2021). Ecologists have yet to grapple with the associated ethical and environmental ramifications of these praxes, but they should note work from other disciplines highlighting the environmental consequences of shifts toward computationally intensive cloud-based processing (Lannelongue et al. 2021). Open sharing of best practice, plus standardization of workflows and processing protocols within drone ecology are vital to ensure that the community can develop efficient approaches and reduce excess trial and error. The latter generates significant processing efforts with associated energy use.

### Drone ethics

Public support has been shown as being moderate to strong for drone use within environmental protection programs (81%; US context), higher than for domestic applications (Markowitz et al. 2017), but increased experimentation has led to deeper critical consideration of drone operations from wildlife biologists, conservation scientists (Sandbrook 2015), and critical geographers commenting on ecological work with drones (Millner 2020, Millner et al. 2024). A wide range of operational protocols and policy guidance now exist to guide fieldwork operations and ethics assessment (Vas et al. 2015, Barnas et al. 2020, Raoult et al. 2020, Weston et al. 2020, Mo and Bonatakis 2022, Jackman et al. 2023, Millner et al. 2023). Ecologists have focused on noise disturbance as a major concern, evaluating the physiological and behavioral impacts on nonhuman species (Hodgson and Koh 2016). Generalizing the findings of experiments with many different taxa, there are some straightforward outcomes: Smaller electric drones create less disturbance than larger fuel-powered drones do (Mulero-Pázmány et al. 2017), some species are more sensitive to certain noise profiles than others (Mulero-Pázmány et al. 2017, Bevan et al. 2018), and some drone maneuvers produce more noise than others (i.e., ascending, descending, and hovering; Macke et al. 2024). However, there are nuanced responses (a recent meta-analysis revealed variable avian responses to interactions with drones; Brisson-Curadeau et al. 2025). Duporge and colleagues (2021) urged ecologists to consider the specific sound profile and frequency of drone noise, noting that sound propagation and loss can be changed by environmental factors and atmospheric conditions. Further work by ecologists in this realm is needed particularly to understand the diversity of effects beyond auditory impacts. Brisson-Curadeau and colleagues (2025) summarized how visual factors such as drone color and how the drone is operated (the angle of approach, the speed of flight) all require careful consideration.

## Drone futures in ecology

In Anderson and Gaston (2013), we argued there was a large gap between applications of drone technology in ecology that were then technically possible and the use being made of them. Ten years on, this gap is arguably just as wide, because, despite the breadth of uses that ecologists have made of drones, what is now possible has advanced immensely. In this section, we highlight promising areas for future research using emerging studies from ecology and other disciplines.

### Innovations in drone sensing and vision

Ecological drone science has mapped, tracked and counted a great diversity of organisms in their habitats. Largely, this drone vision has been delivered by downward-looking standard optical sensors during daylight hours. Ecologists can now experiment with a wider variety of sensors, operational settings, or ways of flying a drone to answer new questions about ecological systems.

#### Vision systems

Mirroring the dominance of daytime ecology generally, there is a comparative lack of nocturnal ecological studies with drones. This may relate to the relatively stronger legislative restrictions placed on nighttime drone operations in many countries or to the greater challenge of nighttime flying. However, this gap is at odds with the now widespread recognition of nonhuman animals having alternative visual and perceptual capabilities and also overlooks that a high proportion of life on Earth is night active (Gaston 2019). Vision systems of night active animals often have marked low-light capabilities. Exploiting nonoptical collision avoidance, and as low-light sensor systems become increasingly compact and lightweight, drones could be used better to understand how animals navigate and use low-light environments. Thermography (either stills or video; see also the “Flying cameras for ecological survey” section) offers the most promising approach for nighttime surveying, although it is important to optimize the approach to the target species by adjusting the flying height and camera angle and by considering local climatology (Burke et al. 2019). Initial work exploring drone flight and sensor optimization for nocturnal studies has been undertaken (Bhattarai and Lucieer 2024), but this warrants further development. In nighttime environments, there is also more widespread use of other sensory systems (olfaction, echolocation, infrared detection, electrosensing) beyond vision, so development or adaptation of appropriate nonvisual sensors for drone deployment may provide novel insights. Such nocturnal experiments will warrant drone modifications for nighttime operation and ethics and risk assessment of specific nocturnal risk factors. In daylight monitoring, more attention should be paid to matching the spectral data captured with animal vision systems. For example, canopy level patterns of ultraviolet reflectance arising from variable leaf orientation can influence avian species because of their tetrachromatic vision (Tedore and Nilsson 2019), affecting foraging or predation avoidance. The centimetric resolution of drone data uniquely allow leaf-level spectral variability to be understood within a canopy context, but new sensor models, particularly those that deliver information beyond basic RGB (red, green, blue) optical data should be trialed.

#### Volumetric manoeuvring

The strong bias toward nadir sensing in ecology decomposes the drone's volumetric agility to a rather unimaginative two-dimensional airborne lawnmower, flying back and forth. Exploiting the drone's 3D agility opens a potential to mimic nonhuman viewpoints more closely. Paradoxically, robotics researchers have long exploited nonnadir sensing vision workflows to enable autonomous drone navigation in complex environments (Kangunde et al. 2021), but ecological researchers have not capitalized on these same innovations. This is despite work showing that a wide range of diurnal flying organisms use such visual optical flow cues to navigate complex volumetric environments (Bhagavatula et al. 2011) and the very sensors used to inform autonomous maneuvers being inspired by insects (Davis et al. 2009).

#### Navigation and autonomy

The aerial viewpoint provides an excellent vantage point from which to observe dynamic ecological behavior during key events (foraging, hunting, inter- and intraspecific competition, migration). So far, the major breakthrough with drone-captured data has exploited image-processing workflows for automatic tracking of individuals’ behavior *post hoc* (Torney et al. 2018, Petso et al. 2021). However, there is great promise for drones to act autonomously, adjusting flight paths to watch or follow individuals or groups vicariously. This builds on robotics engineering work where video feeds can be analyzed on the fly, enabling drones to perform autonomous actions, absent from human control (e.g., drones responding to human gestures; Pourmehr et al. 2013). These approaches could be modified to respond to animal behavior cues—for example, locating a specific organism or trait and tracking individuals to learn about behavior. Within agricultural settings, machine-learning-based analysis of thermographic video feeds has been shown to work well for automated segmentation, detection, and tracking of dairy cattle (Bárbulo Barrios et al. 2024), whereas work in the search-and-rescue sphere shows promise for automatic recognition of human bodies in different orientations (Sandino et al. 2020). It is not a major leap to suggest drone data could be used to find and follow all kinds of different species or identify behavioral patterns, perhaps in real time. The major constraints are on grain (the size of the target organism compared with the drone's sensing capability) and speed of movement (rates of change) relative to physical and computational tracking capability. Basic remote-sensing experiments to establish optimal data capture approaches, and advances in signal processing or machine learning approaches for information extraction will both be central to such developments.

#### New sensors and image processing approaches

Over the past decade, the pixel resolution of optical sensors alone has increased by a factor of three, placing a growing burden on drone ecologists to store, process, and manage data volumes. There are hardware and software solutions now available to address such challenges, but these have not yet been trialed within drone ecology. Event cameras are novel instruments that record changes in brightness only in pixels exhibiting change, with microsecond temporal resolution, but to date, these have only been used in drone navigation rather than in ecological data capture (Falanga et al. 2020). Event cameras could enable more accurate tracking of objects with lower dimensionality, potentially providing a useful solution to some tracking approaches highlighted above. Image processing solutions can be found via techniques such as foveation (Silva et al. 2020); although it did not reduce the volume of data captured, it offers a means of reducing redundancy in the processing workflow by coarsening the spatial resolution of image regions that are irrelevant to the application in hand. Integration of such technologies into drone ecology workflows could reduce the data burden significantly.

### New drone mobilities

Most of the past decade has seen aerial experimentation immediately above land or sea surfaces, but there are wider ecological domains warranting exploration with drones, and this may be enabled in an age where drone mobility is diversifying rapidly. In the present article, we imagine future avenues for drone research in the understory, underground, atmosphere, and urban areas.

#### Drone diversity

The past decade has seen a wealth of engineering developments that allow drones to perch (Hang et al. 2019), use robotic arms (Nguyen et al. 2018), and operate in multiple mobilities (e.g., fly and swim or fly and climb; Myeong and Myung 2018); they all offer potential for new ecological experiments in diverse settings. Graule and colleagues (2016) described, for example, a flapping-wing insect-like drone weighing 100 milligrams, whose switchable electroadhesive pad enables attachment or detachment from surfaces. Such (potentially cryptic) technology could be used by ecologists to monitor within insect swarms, canopies, or on the surface of another organism's body. In addition, the now relatively advanced drone delivery systems that have been developed for shopping or medical deliveries (Scott and Scott 2019) have strong potential for ecological applications (e.g., in delivering or collecting samples from remote locations). However, incorporating long-distance flights into ecological studies will require consideration of beyond visual line of sight (BVLOS) operating cases, which are technically, operationally, and legislatively complex (i.e., requiring a special safety case to be made to the relevant aviation authorities; Hartley et al. 2022). In some settings BVLOS operations could be hugely beneficial to ecologists—for example, in forests or remote areas where takeoff and landing positions are limited. BVLOS operations have already been proven in conservation drone applications (Koh and Wich 2012), but broader application and development of operational BVLOS models for ecologists require improved systematic integration of drones into general airspace.

#### Understory

There is a dearth of drone work *within* canopies, despite the understory and volumetric structure of woodlands being a core ecological concern. Within-canopy experimentation with drones could deliver new insights (e.g., viewpoints of canopy architecture according to arboreal species, biomass, and connectivity studies) but is currently complex to achieve because of drones having built-in obstacle avoidance and the understory being GPS denied. There are opportunities for more agile handling of drones within canopies, including vision-based navigation leveraging monocular, stereo, or fisheye cameras (Lu et al. 2018). Operational systems with such capabilities have yet to be released commercially, but once they reach the market, this would be an area ripe for ecological exploration.

#### Underground

**Figure 4.**
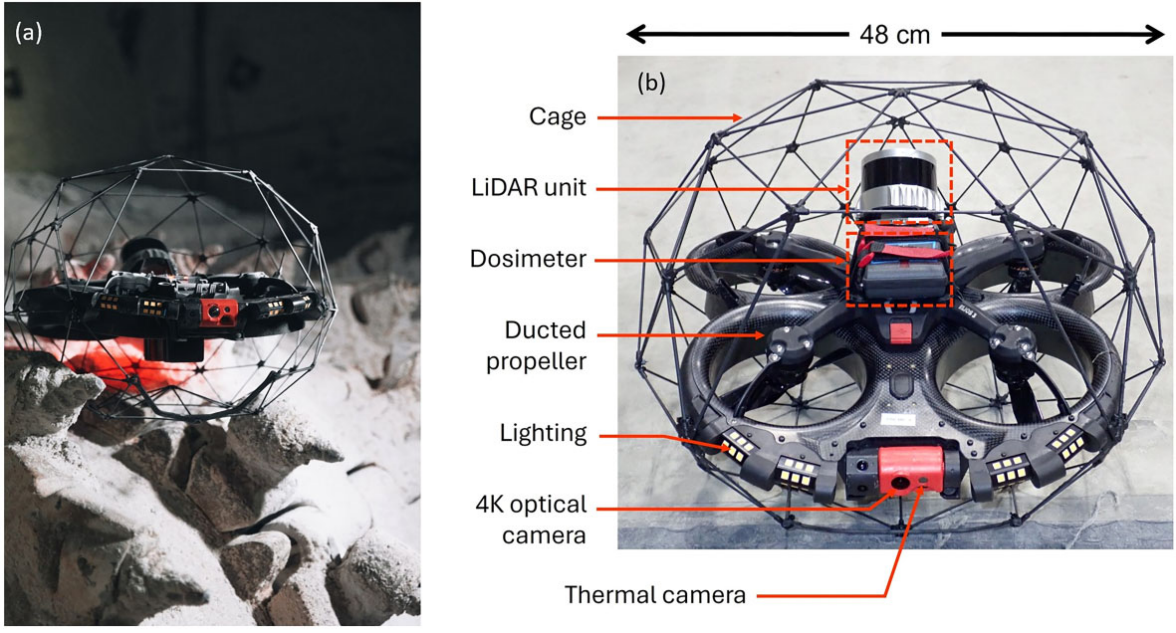
(a) The Elios drone. Designed for underground inspections and fitted with a collision-resilient cage, this could be adopted by ecologists for mapping subterranean environments (Image: Flyability SA). (b) Elios is equipped with optical and thermal cameras and a LiDAR unit (Image: the Idaho Environmental Coalition; Young et al. 2024).

Cave environments are GPS denied and structurally complex but offer unique natural experimental laboratories for testing ecological questions (Mammola et al. 2020). Uses of drones underground to date have tended to focus on tackling issues of human safety, for example, exploration within confined, contaminated environments (Young et al. 2024). New subterranean ecology-focused drone experiments could deliver insights about structure, connectivity and form (including in areas off limits for humans). There is also scope for such surveys to reveal the composition and dynamics of subterranean ecological communities. Technology exists already in systems such as the Elios drone (figure 4), which is equipped with LiDAR or thermal sensors and has a collision-resilient cage. This system is widely used in applications such as mineshaft inspection but has not yet been trialed in underground ecology. Furthermore, developments in the design of amphibious drones for other applications could be beneficial in such settings where water bodies might otherwise inhibit access to cave systems (Evangeliou et al. 2023).

#### Aerobiology

Aerial dispersal is a key mechanism shaping biodiversity but remains poorly understood. Scientists working in sensitive environments have identified that aerial transport of microorganisms, spores, and propagules is a key dispersal process (Pearce et al. 2016). Characterizing the nature and direction of flows and interactions is crucial for understanding processes and mitigation. There is limited evidence that aerobiological sampling has been trialed from drones within different atmospheric layers (Pearce et al. 2016). Recent experiments have however shown that aerial samplers (used widely in piloted aircraft) can be deployed on drones to characterize the undersampled boundary layer (Liu et al. 2021). Wider investigation of this is warranted.

#### Urban

Although urban environments are not GPS denied, they have in most countries been *policy denied*, where drone use is restricted to ensure safety of people and infrastructure (because of collision risks, as well as privacy laws). In consequence, urban ecology remains a gap in drone ecology literature. The smallest consumer drones (less than 250 grams TOM; figure 1) now enable new urban ecology experiments. Although a concern might be that their small size compromises accuracy, studies such as those by Mugnai and colleagues (2022) have shown that their sensors are quite capable and sufficient for most ecological investigations. In addition, multimobility drones such as those that can fly and perch (Thomas et al. 2016) or fly and climb walls (Mahmood et al. 2021, Ottaviano et al. 2022) offer urban ecologists a wider range of possibilities for surveying in urban environments with complex vertical geographies.

### Critical drone ecology

So far, ecologists have engaged drones as tools for mapping, collecting samples, or monitoring animal behavior. But as the uses of lightweight drones expand, we urge ecologists to become more critical in their engagements with this technology, considering that more than ecological philosophy is at play when drones are in use. Commercial drones are complex instruments that have evolved from militaristic technology, a history that is no more sharply in view than when drones are leveraged in ecological conservation to kill—for example, in aerial baiting for rat poisoning in the Galapagos islands (Marris 2019). We urge ecologists to build on critical drone work from other disciplines, engaging the drone as more than a camera on high (Fish and Richardson 2021), accepting that drones can “give and take life, empower and oppress, and open up new potentials for justice, as well as enclose and control” (Fish and Richardson 2021, p. 4). In this section, we highlight some areas that need to receive critical consideration by ecologists. Most demand interdisciplinary approaches (advocated by Jackman et al. 2023).

#### Environmental impacts

Ecologists need to consider the life-cycle impacts of drone-centered experiments and how these are balanced against the societal or environmental benefits of the research. Drone construction uses environmentally damaging ingredients—for example, lithium (batteries), copper (cables), and aluminum or plastic (frames). There are also footprint (electricity, water) considerations related to information extraction from the sizeable spatial data sets generated, particularly if cloud services are used to process data or computationally expensive machine learning workflows are leveraged (Lannelongue et al. 2021). Of course, materials for drone construction are constantly evolving, and the future will undoubtedly include biodegradable drones (Wiesemüller et al. 2022). We suggest that ecologists have a role to play in designing and testing more environmentally sensitive drone designs.

#### Community engagement

Table 2 outlines how drones have been trialed in seed dropping for forest recovery. The role of drones in ecosystem restoration and conservation is ripe for exploration by ecologists as rewilding initiatives grow in popularity. Despite the identified fallacies of seed-dropping methods so far trialed, drones have been shown to be capable devices for empowering communities who are often otherwise marginalized in restoration or conservation projects (Fish 2022b). Some argue that the machines themselves can help to resolve tensions among communities, technologies, and nonhumans (Muashekele et al. 2022). More work involving ecologists is needed in this emerging area.

#### Multi-robot collaborations

Drone swarms are either represented as icons of dystopian futures (e.g., drone pollination; Rehna and Inamdar 2022) or as a source of visual entertainment (e.g., in public light shows). Tangible ecological uses for swarms mostly lie somewhere between these extremes—for example, for wildfire monitoring (Saffre et al. 2022)—although ecological experimentation with swarms has been minimal to date. In engineering, rapid progress is being made toward autonomous capabilities within complex or cluttered environments and swarm coordination without external facilities (Zhou et al. 2022), which paves the way for swarms to be used in capturing ecological data (especially if swarms can be flown out-of-the-box and if legislative restrictions ease as swarm safety is proven). Furthermore, although we have so far restricted our discussion to flying robots, we bring it to a close by highlighting other robotic developments such as the peristaltic earthworm-inspired soil robot designed by Das and colleagues (2023). Aerial drones could deploy earthworm drones, allowing the linking of, for example, subsurface ecology (measured by earthworm robots) with surface trait information gathered aerially. Ecologists should consider how drone swarms and multirobot collaborations could become a future source of ecological volumetric information.

## Conclusions

Ten years on from Anderson and Gaston (2013), drones have become a well-established addition to the ecologist's toolbox and, with reductions in weight and improvements in capability, are now in a strong position to become part of the field equipment routinely carried (such as binoculars or photographic equipment). Drone ecology has clearly provided insights that otherwise would not have been possible or that could not have been achieved as effectively or efficiently. However, there does remain a substantial gap between ways in which ecologists use drones and what is possible. Exploiting this gap has in some ways become much easier because automation has reduced the need for ecologists to become proficient in hands-on flying of drones, allowing data collection to become the main focus. But this new dawn of drone ecology will require ecologists to consider deeper social, environmental, and ethical issues arising from drone praxes. We have highlighted ways in which the next decade of drone experimentation may drive new volumetric ecological understanding and the need for vigilance regarding ethics of drone use. We refer readers to table 3, which summarizes the main opportunities we foresee during the next decade of drone ecology, along with barriers to uptake and recommendations for pathways forward. This evidences how engaging drone technology during its next evolutionary phase will undoubtedly require ecologists to reach beyond their scientific boundaries and embrace interdisciplinary work.

**Table 3.** Emerging opportunities within drone ecology for the next decade, with barriers to uptake and pathways forwards identified.

Opportunities for drone ecology	Barriers to uptake	Pathways forward
Improved exploration of nocturnal ecology	Nighttime flying carries greater operational complexity with added legislative restrictions. Sensor capabilities and calibration in low-light conditions remain challenges for some applications.	Platform technological capacity has been proven. Ecologists should trial new sensing approaches beyond the *status quo* of thermography and visual sensing. This area will benefit from more diverse experimentation to push the frontiers.
Exploit the drone's capability for 3D maneuvers	Use cases for autonomous robotic operations are largely non-ecological (e.g., search and rescue, consumer delivery). 3D manoevering offers great potential for real-time following of mobile organisms (e.g. imagine the behavioural understanding that could be unlocked by tracking insects in flight), but users should consider grain size constraints (i.e. size of target organisms compared with sensing capability) and speed of movement (i.e. rates of change) relative to computational tracking capability.	Great potential for new insights into understory, underground and in tracking moving organisms (including developing ecologically optimized autonomy). Improved collaboration between ecologists, drone designers and sensor engineers is needed to realize applications for biology-inspired design. Integration of machine learning workflows into the drone's control system, would enable tracking of objects or species in real time, autonomously. Work is needed at the interface of systems engineers, control theorists and ecologists to push this boundary. Sensor optimization work is needed to improve understanding of drone control and data collection in challenging ecological settings.
Urban applications of drone surveying and sampling	Previously underexplored because urban areas were policy denied for heavier drones. With the recent advent of camera-equipped drones with a takeoff mass of less than 250 grams, a major remaining barrier is a privacy one. New multimobility drones have emerged from engineering fields that could benefit urban ecological studies.	Explore whether advanced machine-learning and image-processing workflows can remove human subjects from images or human voices from acoustic recordings prior to data processing or sharing to maintain privacy. Ecologists should engage with new multimobility drones to scope use cases in urban systems.
Broader exploitation of beyond visual line of sight capabilities	Proven in conservation drones and non-ecological use cases. Major barriers are technical (e.g., endurance) and legislative (e.g., policy restrictions on long-distance flights).	Improved integration of drones into airspace management systems is needed to pave the way for more widespread use. Ecologists should trial BVLOS in new settings where it can be safely demonstrated.
Ecologically sensitive drone science	The environmental life cycle of drone technology is poorly explored. Ecologically-inspired designs (e.g., biodegradable drones) are new.	Knowledge exchange between ecologists and engineers.
Drone data storage, sharing and processing impacts	Workflows for scoping this are available from other fields (genetics and biological computation).	Scope the life-cycle impacts of drone data using existing tools. Identify ways of improving efficiency in data processing and sharing.
Community engagement	Largely engaged from a cultural geography or ethics perspective to this point.	Drone ethics extends beyond impacts on target organisms. Ecologists could engage more with the social implications of drone-based work across all use cases, and learn from the work done by humanities scholars.
Multirobot collaborations (swarms and more)	The control systems and aviation safety case for swarm optimization have been proven for non-ecological drone demonstrations (e.g., in entertainment spheres). The challenge is in the segue to ecological applications. New robotic forms (e.g., soil burrowing robots) have not yet been integrated with other drone technology but this offers a tantalizing ecological opportunity.	Perform a scoping study involving ecologists and robotics engineers to imagine ripe areas for swarm testing and multirobot collaborations. Ecologists should evaluate the opportunities offered by the broad suite of operational autonomous robots.
Integration of event cameras and drones	Lack of proven testing in aerial domain. Will probably deliver greatest insights from hovering experiments where subjects in the scene are mobile, but the drone is not.	Experimentation and reporting.
Understory ecology	Could leverage existing vision-based navigation (e.g., from monocular, stereo or fisheye cameras), or use drone-mounted lidar. Obstacle avoidance (now standard on commercial drones) will prevent easy navigation of the understory. Self-built drones without collision avoidance fail-safes will offer improved understory agility, but at the cost of needing a competent pilot, perhaps with first-person view flying skills.	Experimentation and reporting.
Matching drone spectral data with animal vision systems	Sensors are available that permit drone sensing beyond RGB. The challenge is in integrating new sensors on drone platforms and calibrating them in the operational domain.	Experimentation and reporting.
Animal behavior	Tracking of individuals and groups is possible from aerial video streams. Autonomous tracking in flight is the next step.	Experimentation and reporting.
Demonstration of cage-mounted drones for ecological survey in challenging environments	Proven capability via technology such as the Elios drone (figure 4), but not yet trialed in ecological experiments. Great scope for deployment and exploration in subterranean ecological investigations.	Experimentation and reporting.
Multimobility drone tests for ecological experimentation, particularly in urban systems	Drones that can perform maneuvers in multiple mobilities (e.g., amphibious drones, drones that can perch and fly or those that can fly and climb) are new and use cases are relatively untested, particularly within ecology.	Experimentation and reporting.
Boundary-layer ecological studies or aerobiological investigations	Lacking a strong ecological proof of concept despite drone technology being at a sufficient readiness level. Policy governing scientific access to elevations outside of normal drone operation range (e.g., up to 100 meters or 400 feet above ground level) would probably require a special safety case to be made.	Experimentation and reporting.

## Footnotes

1 Covering thousands of articles; Web of science search 22 October 2024 for related terms *UAV AND ecology*, 560 results; *drone AND ecology*, 752 results; *drone AND vegetation*, 1386 results; *drone AND forestry*, 533 results; *drone AND conservation*, 917 results; *drone AND restoration*, 328 results; many ecological papers featuring drone methodologies in ecological investigations do not appear in these searches.

## Author Biography

Karen Anderson (karen.anderson@exeter.ac.uk) and Kevin J. Gaston are affiliated with the Environment and Sustainability Institute at the University of Exeter, in Penryn, Cornwall, England, in the United Kingdom. Felipe Gonzalez is affiliated with Robotics and Autonomous Systems, at the Queensland University of Technology, in Brisbane, Queensland, Australia.
